# Characterization of the complete plastome of medicinal plant *Saururus chinensis* (Saururaceae)

**DOI:** 10.1080/23802359.2019.1667909

**Published:** 2019-09-25

**Authors:** Lei Jin, Jin Yang, Changkun Liu, Mengling He, Hanjing Yan

**Affiliations:** a College of Traditional Chinese Medicine, Guangdong Pharmaceutical University, Guangzhou, Guangdong, China;; b Key Laboratory for Plant Diversity and Biogeography of East Asia, Kunming Institute of Botany, Chinese Academy of Sciences, Kunming, Yunnan, China;; c School of Life Science, Yunnan University, Kunming, Yunnan, China;; d Key Laboratory of State Administration of Traditional Chinese Medicine for Production & Development of Cantonese Medicinal Materials, Guangzhou, Guangdong, China

**Keywords:** *Saururus chinensis*, Complete plastome, Phylogenetic analysis

## Abstract

*Saururus chinensis* is an important medicinal plant in Southeast Asia. Here, we determined the first complete plastome of *S. chinensis* using high throughput Illumina sequencing technology. The *S. chinensis* plastome is 161,494 bp in length and presents a typical quadripartite structure consisting of one large single-copy region (LSC, 88,863 bp), one small single-copy region (SSC, 18,679 bp), and a pair of inverted repeat regions (IRs, 26,976 bp each). The phylogenetic analysis robustly supports that *S. chinensis* is sister to the group including the *Saruma henryi*, *Asarum sieboldii*, *Piper kadsura*, *Piper cenocladum.*

*Saururus chinensis* (Loureiro) Baillon, a perennial herb commonly called Chinese lizard’s tail, belongs to the family Saururaceae. The species is endemic to China, India, Japan, Korea, Philippines, and Vietnam (Angiosperm Phylogeny Group 2016). It has been traditionally used as a medicinal herb for the treatment of edema, jaundice, gonorrhea, and several inflammatory diseases (Yoo et al. 2008; Yu et al. 2008; Chang et al. 2011). However, there are no studies on the genome of *S. chinensis* before the current work, which seriously hinders the management of the germplasm resource and conservation of this medically important plant. Here, we sequenced and assembled the complete plastome of *S. chinensis*.

Specimen and leaf tissues of *S. chinensis* were collected from the Botanical Garden of Kunming Institute of Botany, Chinese Academy of Sciences (25°8′21′′N, 102°44′25′′E). Voucher specimen (Y. Ji 2017086) was deposited in the Herbarium of Kunming Institute of Botany, Chinese Academy of Sciences (KUN). Silica gel dried leaf tissues were used to extract total DNA by the modified CTAB method (Yang et al. 2014). Purified DNA was sheared to fragments with an average length of 500 bp to construct a shotgun library. The library was sequenced on Illumina HiSeq 2000 system. The complete plastome of *Asarum sieboldii* (GenBank Accession No. MG551543) was downloaded as the reference. We assembled the plastome following the method described by Jin et al (2018). The assembly was edited and annotated according to the reference in Geneious V10.2 (Kearse et al. 2012). Start and stop codons and intron/exon boundaries for protein-coding genes were checked manually. The plastome sequence of *S. chinensis* was deposited in the NCBI GenBank database (MN263891).

The *S. chinensis* plastome is 161,494 bp in length and presents a typical quadripartite structure consisting of one large single-copy region (LSC, 88,863 bp), one small single-copy region (SSC, 18,679 bp), and a pair of inverted repeat regions (IRs, 26,976 bp each). The plastome encodes 114 unique genes (80 protein-coding genes, 30 tRNAs, and 4 rRNAs). Among these unique genes, 12 protein-coding genes (*atp*F, *ndh*A, *ndh*B, *pet*B, *pet*D, *rpl*16, *rpl*2, *rpo*C1, *rps*12, *ycf*3, *clp*P, and *rps*12), and 6 tRNAs (*trn*A-UGC, *trn*G-UCC, *trn*I-GAU, *trn*K-UUU, *trn*L-UAA, and *trn*V-UAC) contain at least one intron. The overall G/C content in the *S. chinensis* plastome is 38.50%, and the corresponding values for LSC, SSC, and IR regions are 37.00%, 32.60%, and 43.00%, respectively.

The relationships among Piperales species were reconstructed based on complete plastome DNA sequences. We used RAxML (Stamatakis 2014) with 1000 bootstraps under the GTRCAT substitution model to reconstruct the phylogenetic tree. *Drimys granadensis* (Winteraceae) was used to root the tree. The phylogenetic analysis robustly supports that *S. chinensis* is sister to the group including the *Saruma henryi*, *A. sieboldii*, *Piper kadsura*, *Piper cenocladum* (Figure 1). The complete plastome of *S. chinensis* will provide a useful resource for the conservation genetics of this species as well as for the phylogenetic studies of Saururaceae.

**Figure 1.**
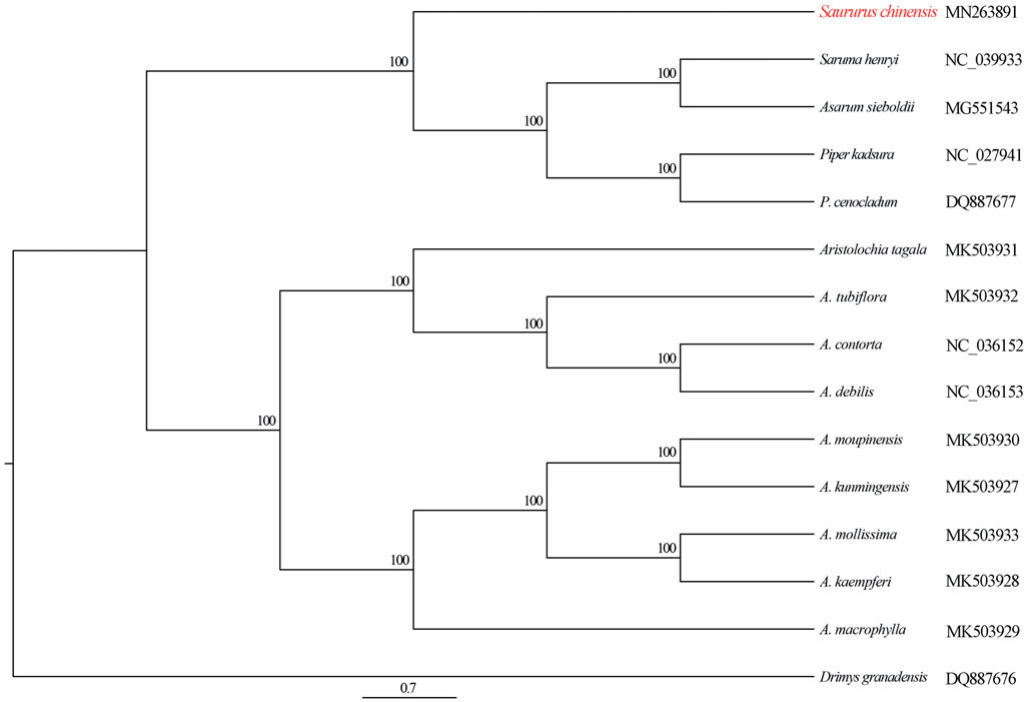
Maximum-likelihood (ML) tree was reconstructed based on 15 complete plastomes. The numbers represent the bootstrap values.
